# Role and Mechanisms of Phytochemicals in Hair Growth and Health

**DOI:** 10.3390/ph16020206

**Published:** 2023-01-30

**Authors:** Periyanaina Kesika, Bhagavathi Sundaram Sivamaruthi, Subramanian Thangaleela, Muruganantham Bharathi, Chaiyavat Chaiyasut

**Affiliations:** 1 Innovation Center for Holistic Health, Nutraceuticals, and Cosmeceuticals, Faculty of Pharmacy, Chiang Mai University, Chiang Mai 50200, Thailand; 2 Office of Research Administration, Chiang Mai University, Chiang Mai 50200, Thailand

**Keywords:** hair loss, alopecia, phytochemicals, hair health, hair growth stimulation

## Abstract

Hair health is associated with personal distress and psychological well-being. Even though hair loss (alopecia) does not affect humans’ biological health, it affects an individual’s social well-being. So, treatment for hair problems and improving hair health are obligatory. Several pharmacological and cosmeceutical treatment procedures are available to manage hair loss and promote growth. Several factors associated with hair health include genetics, disease or disorder, drugs, lifestyle, chemical exposure, and unhealthy habits such as smoking, diet, and stress. Synthetic and chemical formulations have side effects, so people are moving towards natural compounds-based remedies for their hair problems. The history of using phytochemicals for hair health has been documented anciently. However, scientific studies on hair loss have accelerated in recent decades. The current review summarizes the type of alopecia, the factor affecting hair health, alopecia treatments, phytochemicals’ role in managing hair loss, and the mechanisms of hair growth-stimulating properties of phytochemicals. The literature survey suggested that phytochemicals are potent candidates for developing treatment procedures for different hair problems. Further detailed studies are needed to bring the scientific evidence to market.

## 1. Introduction

The history of alopecia areata (AA) starts approximately 1500 before the common era (BCE), but scientific studies on hair loss have accelerated in recent decades in terms of publications [1]. Even though hair is not important for humans in terms of biological protection, hair loss has significant social, psychological, and emotional impacts on everyone. Therefore, treatment for hair loss and improving hair health is obligatory.

Humans have ~100,000 scalp hair shafts with varying degrees of hair growth, and the average life cycle of a hair shaft is ~3.5 years, with a growth rate of 0.05 inches per month. The hair has three regions, such as medulla (innermost layer, developed from transparent cells and varies among hair types), cortex (middle layer, which provides strength to the hair shaft, composed of keratin protein), and cuticle (outer layer). The hair follicle can be divided into the lower, middle (isthmus), and upper (infundibulum) segments. The lower segment includes the bulb and suprabulb regions. The middle segment of the hair follicle includes the region covering the arrector pili muscle insertion to the opening of the sebaceous gland duct. The upper segment of the follicle includes the region between the opening of the sebaceous gland duct and the follicular orifice [2].

Anagen (growth phase, lasts for 2 to 7 years), catagen (exponentiation, lasts for 2 to 4 weeks), and telogen (resting phase, lasts for 3 months) are three major phases of the hair growth cycle. The amount of scalp hairs may change based on the anagen (85 to 90.6%), telogen (10 to 15%), and catagen (1 to 2%) phases. Pigmented hair shafts are produced in the anagen phase, and the follicle achieves its maximum length and volume. During the catagen phase, the epithelium of the lower follicle breaks and grows up with the papilla until it lays below the bulge zone, establishing the club hair. Telogen is a quiescence phase of the hair cycle, characterized by the reduction of proliferation and biological activity of hair follicles [2,3,4].

Hair color, density, hair fiber curvature, and diameter are affected by the aging of hair, which overall contribute to the appearance and manageability of hair [5,6]. Numerous factors affect hair health and the hair growth cycle. Heavy metals (thallium, mercury, arsenic), toxins (Botulinum, *Podostroma cornu-damae*), drugs, medications, genetics, stress, smoking, menopause, lifestyle, and diet are some of the major factors associated with hair health [7,8,9,10,11,12,13,14].

Various drugs and treatment strategies were used to treat hair loss. Minoxidil was the first drug approved by the FDA to treat hair loss [15,16]. 5α reductase inhibitors (e.g., Finasteride, Dutasteride) are also used to treat male androgenetic alopecia [17,18]. The combination of finasteride and minoxidil treatment significantly improved hair health, and the combination therapy is more effective than a single treatment procedure. Furthermore, hair transplantation [19] and cell therapy [20] are effective treatments for hair loss.

Many phytochemicals include epigallocatechin gallate (EGCG), caffeine, capsaicin, procyanidin, onion juice, pumpkin seed oil, rosemary oil, saw palmetto [21,22], red ginseng extract [22], curcumin, garlic gel, and other natural products such as amino acids, marine proteins, melatonin, vitamins, and zinc [21], were reported to have hair growth-stimulating property [21,22]. Recently, researchers have been interested in herbal-based nanomedicine for hair health [23]. Furthermore, several mechanisms have been proposed and proved for the hair growth-promoting properties of phytochemicals [24,25,26,27,28,29,30].

The current manuscript summarizes the factors affecting hair health, types of hair loss, treatments for hair loss, the role of phytochemicals in managing hair loss, and proposed mechanisms associated with hair loss prevention/growth-stimulating properties of phytochemicals. This concise review may provide basic information on the role of phytochemicals in hair health.

## 2. Types of Alopecia

Alopecia can be classified based on the cause and appearance and rarely by gender. Androgenetic alopecia (AGA), telogen effluvium (TE), alopecia areata (AA), and scarring alopecia (SA) are the common types of hair loss [31].

AGA is an androgen-dependent hair loss. About 58% of men may be affected by AGA. Hair loss starts with bitemporal hairline decline and thinning at the vertex and frontal-parietal regions. About 33% of Caucasian women aged 70 or older may be affected by AGA. Postmenopausal and genetically susceptible women are mainly more sensitive to AGA [31].

TE is a nonscarring form of hair loss, a scalp disorder exemplified by excessive hair shedding [32]. The changes in the hair cycle could cause TE, which could be acute or chronic hair loss. Older women are more susceptible to acute TE attributed to drugs, childbirth, and thyroid-related diseases [33], even though age’s influence on TE is unclear. The incidence of TE in children was about 2.7% in Southeast Nigeria [34]. Telogen gravidarum, nutrition, zinc, iron deficiency, genetics, chronic renal and hepatic failure, syphilis, early AGA, infection, stress, and malignancy are some factors that cause TE [33]. There are five types of TE based on the hair follicle cycle: immediate and delayed anagen release, immediate and delayed telogen release, and short anagen syndrome [35]. High fever, surgical trauma, starvation, and hemorrhage are the triggers for acute TE. The hair loss may occur after two to three months of the triggering event. Not all cases have the triggering event in their life. So, the cause of TE is still not yet understood completely [33,35]. The diagnosis of acute TE can be conducted by history and examination. Acute TE is a self-limiting biological event, so if the hair loss stops spontaneously, no further treatments are needed. Nevertheless, if hair loss continues (Chronic TE), medical evaluations are needed to find the cause and proper treatment [31].

AA is a nonscarring and defined hair loss due to the premature conversion of the anagen to telogen phase in hairs. Family history and autoimmune conditions are the major cause of AA; other than that, no specific cause of AA has been defined yet [36]. A higher incidence of AA has been observed in the female population. The etiology of AA is elucidated completely. The activation of immune cell androgen and estrogen receptors during pregnancy, X chromosome-mediated innate immune response and immune tolerance, and maternal micro-chimerism of fetal immune cell lines are associated with the etiology of AA. The average age of disease inception varied based on ethnicity and gender. Female patients with autoimmune diseases are reported for the incidence and progress of AA compared to their male equivalents [37].

SA is irreversible hair loss and is commonly found in European Caucasian women. SA is divided into two subgroups such as primary SA and secondary SA. In primary SA, hair follicles have microscopic inflammation and damage the follicular epithelium without affecting the interfollicular reticular dermis. Primary SA is associated with chronic cutaneous lupus erythematous (an autoimmune condition), pseudopelade of Brocq, lichen planopilaris (inflammation in hair follicles), folliculitis decalvans (chronic inflammation of hair follicles and scalp), and dissecting folliculitis. The underlying disease that causes primary SA could be predicted by knowing the type of inflammatory infiltrates around hair follicles. In secondary SA, hair follicle damage is related to a non-follicle-directed cause [38]. Exogenous factors such as trauma, endogenous infiltrative, and inflammation cause secondary SA [39]. Central centrifugal cicatricial alopecia (CCCA) caused by hair treatments using chemicals and irons is also considered secondary SA [31], and its incidence is associated with diabetes mellitus [40].

## 3. Factors Affecting Hair Health

**Figure 1:**
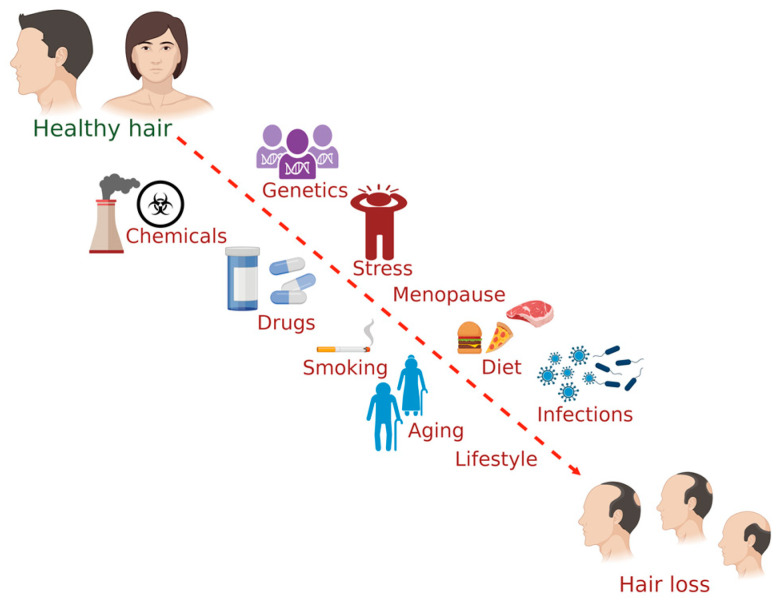
The common factors influencing hair health in humans. (The figure was created using BioRender.com; accessed on 17 November 2022).

Several factors affect hair health, including genetics, chemical exposure, lifestyle, smoking, drugs, stress, infection, and menopause. Evidence for some of the influencing factors of hair health has been detailed in this section (Figure 1).

### 3.1. Diet

Consumption of nutritional supplements such as micronutrients (vitamin A, vitamin E, and selenium) in excess leads to hair loss [41]. Toxic dose exposure to selenium shows symptoms of hair loss, fatigue, foul odor (garlic breath), changes in nails (discoloration, brittleness), nausea, and vomiting. MacFarquhar et al. [42] analyzed the outbreak of acute selenium poisoning due to the consumption of a liquid dietary supplement, which contained 200 times higher than the labeled concentration of selenium. About 201 patients with selenium poisoning showed adverse effects; among them, 140 patients showed hair loss [42]. *Lecythis ollaria* Loefl. nuts (*L. ollaria*; paradise nuts) contain a high concentration of selenium (7 to 12 g of selenium per kg dry mass of *L. ollaria* nuts) in the form of seleno-cystathionine [43]. Two healthy women who consumed a handful of *L. ollaria* nuts showed symptoms of selenium poisoning. Hair loss started for 38-year-old women twelve days after the consumption, and 46-year-old women showed hair loss two weeks after the consumption [44]. A recent study reported that high-fat diet-fed mice showed hair thinning by reducing the hair follicle stem cells compared to the standard diet-fed mice and stated that obesity accelerated the thinning of hair and leads to hair loss [45].

Carbohydrates also affect hair health. The consumption of simple sugar-containing processed foods is one of the indirect factors associated with hair loss. Simple sugar stimulates sebum secretion, and even though sebum is good, excess secretion facilitates microbial growth in the scalp, which further causes irritation and inflammation [46,47]. High sugar intake impacts hair loss by activating the polyol pathways [48,49,50]. A recent study revealed that consuming sugar-sweetened beverages is linked with a high risk of male pattern hair loss in young men [51].

### 3.2. Chemical Exposure

Exposure to heavy metals such as mercury and thallium is a highly associated risk factor for alopecia. Studies have evidenced the toxic effects of those chemicals on hair [52,53]. Case studies report the toxic exposure of arsenic, botulinum toxin, boric acid, Podostroma cornu-damae, synthetic opioid MT-45, and vitamin A. A case study reported heavy metal (arsenic and mercury) poisoning in a 51-year-old man showing anorexia, dizziness, fever, hair loss, and rash after the topical use of Chinese homeopathic medicine for anal fistulas [52]. Sharquie et al. [54] evaluated five patients (gender: male; age range: 10-32 years old) with thallium poisoning due to consuming cakes laced with thallium. All the patients showed symptoms of thallium poisoning, including anagen hair loss that started in the 2nd and 3rd week after thallium exposure [54]. The reported minimum lethal dose of thallium was 10 to 15 mg per kg [55]. A study reported that 1 week after thallium poisoning, three out of five patients showed profound hair loss [53].

### 3.3. Drugs

A retrospective hospital-based study analyzed many cases of poisoning in Sri Lanka caused by the intake of plants and mushrooms that contain toxic substances [56]. *Gloriosa superba* L. (*G. superba*) contains high levels of colchicine, an alkaloid drug. Colchicine is used in the treatment of amyloidosis, familial Mediterranean fever [57], Behçet’s disease [58], and gout [59]. It has a narrow therapeutic–toxicity window and variability in the level of tolerance among individuals [60]. The toxicity risk is dose-dependent, and intake of <0.5 mg per kg is reported with no mortality rate, intake of 0.5–0.8 mg per kg is reported with a 10% mortality rate, and intake of colchicine >0.8 mg per kg leads to 100% mortality rate [60]. The symptoms of colchicine toxicity consist of 3 phases. Gastrointestinal symptoms, diarrhea, leukocytosis, and reduced blood volume occur during the 1st phase (during the first 24 h after ingestion of a toxic colchicine dose). Bone marrow hypoplasia, leukopenia, and possible multiple organ failure occur during the second phase (from 2nd to 7th day). Renewal of the bone marrow activity, rebound of leukocytosis, recovery from multiple organ failure, and the onset of alopecia in the 3rd phase (from the 2nd week onwards is the recovery period). Hair growth starts after 3–12 weeks [61,62].

A male (age: 26 years old) started losing hair on the 9th day after consuming *G. superba* (2 tubers) [63]. A case study reported a child (gender: female; age: 3 years old) with acute colchicine intoxication showed symptoms of multi-system organ failure followed by alopecia, which started on the 2nd week after consuming 20 to 25 pills (a toxic dose of about 0.9 mg per kg) [64]. A case study reported that a female (age: 17 years old) attempted suicide by consuming 40 pills of colchicine (40 mg) which caused severe pancreatitis and bicytopenia, followed by anagen effluvium, which started one week after the colchicine poisoning [65]. Alaygut et al. [66] evaluated 17 pediatric cases with colchicine intoxication until their recovery as an observational case series study during January 2010–2012 at the Pediatric Intensive Care Unit of Cumhuriyet University Faculty of Medicine, Turkey. Among the 17 patients, only 1 patient (gender: female; age: 16 years old) showed total alopecia who ingested colchicine (0.88 mg per kg) on attempting suicide [66].

### 3.4. Diseases or Disorders

Abnormal skin conditions such as atopic dermatitis, dandruff/seborrheic dermatitis, psoriasis, and tinea capitis affect hair health. The hair samples of atopic dermatitis patients (*n* = 8; age range = 24 to 25 years old) showed thick scales and torn cuticular edges, which were examined using atomic force microscopy. The roughness of the hair of atopic dermatitis patients was higher than that of healthy adults (*n* = 15; age range = 20 to 54 years old) [67]. Plozzer et al. [68] examined the hair obtained from psoriasis patients (*n* = 39; age range = 24 to 74 years old) and healthy individuals using scanning electron microscopy. The altered hair conditions, such as abraded cuticular surface and cuticular breakage observed only in the hair samples obtained from the psoriasis patients [68]. Similarly, Kumar et al. [69] examined the hair obtained from psoriasis patients (*n* = 30; age range = 12 to 67 years old) and healthy individuals (*n* = 15; age range = 18 to 62 years old) using scanning electron microscopy. Hair thinning and loss in the psoriatic plaques are due to hair dystrophy (alteration in hair cuticles such as ragged appearance and roughness) and micro pits [69]. The lesioned hairs obtained from the psoriasis patients (*n* = 15; age range = 8 to 67 years old) showed increased macro pits, the roughness of the cuticle, and scale thickness compared to that of the healthy adult individuals (*n* = 15; age range = 10 to 58 years old), which was examined using atomic force microscopy [70]. Kim et al. [71] analyzed the hair shafts from psoriasis patients (*n* = 14; age range = 24 to 75 years) and seborrheic dermatitis patients (*n* = 28; age range = 19 to 78 years) in comparison with the hair samples obtained from healthy adult individuals (*n* = 50; age range = 21 to 60 years). The hair samples of all psoriasis patients included in the study showed macro pits, while only 4% of seborrheic dermatitis patients showed macro pits. Scale thickness of the hair samples was common among psoriasis and seborrheic dermatitis patients [71]. Seborrheic dermatitis is associated with premature hair loss [72]. Alterations in the hair, including corkscrew hairs, comma hairs, or zigzag hairs, were observed in patients with tinea capitis (fungal infection) [73].

### 3.5. Smoking

An observational study stated that smoking is associated with premature graying of hair in both men and women and baldness in men [74].

Su and Chen [12] studied the association between smoking and androgenetic alopecia by conducting a community-based cross-sectional survey (10th April and 12th June 2005) among men (*n* = 740; ≥40 years old) living in Tainan County, Taiwan. Based on the survey, the family history of androgenetic alopecia among 1st and 2nd degree relatives is associated with an increased risk of moderate or severe androgenetic alopecia. Besides the family history and age, the risk factors for androgenetic alopecia include cigarette smoking [12]. A cross-sectional study conducted by Chaudhry et al. [75] with men (*n* = 398; ≥36.50 ± 12.11 years old) concluded that smoking is the risk factor for baldness. A recent study showed that premature graying and hair loss prevalence is higher in smokers than in nonsmokers [76].

### 3.6. Genetics

Chumlea et al. [9] investigated the association between the expression of androgenic alopecia in male individuals (from the general community) and their family history of androgenic alopecia. They stated that family history and age are the high-risk factors for the onset of male pattern hair loss. Other risk factors might include hair loss in the father and hair loss in the mother or maternal grandfather [9]. Lukasik et al. [10] studied the association between the family history of androgenic alopecia in females and the onset of female pattern hair loss in women from the Polish population. Family histories of female individuals (*n* = 111 unrelated patients) with androgenic alopecia and female individuals (*n* = 129) with no hair thinning were analyzed. It stated that a family history of maternal hair loss is a high-risk factor for the onset of female pattern hair loss in women. Other high-risk factors might include a family history of androgenic alopecia in grandparents [10].

### 3.7. Stress

Stress is a risk factor for hair loss and inhibition of hair growth. Chronic or acute stress is a primary inducer of hair growth disorder, namely, telogen effluvium; it acts as an aggravating factor for hair growth disorders such as androgenetic alopecia and alopecia areata [77]. Adrenal glands release stress hormones induced by stress signals. Cortisol is a stress hormone released in humans during stress conditions, and corticosterone is a stress hormone in rodents. A recent study stated that corticosterone inhibits the activity of hair follicle stem cells by suppressing the expression of the growth arrest-specific 6 (*gas6*) gene, thereby extending the hair follicles’ resting phase. Corticosterone-induced inhibition of hair follicle’s stem cell activity recovered by restoring the expression of the *gas6* gene [11].

### 3.8. Menopause

Chaikittisilpa et al. [13] conducted a cross-sectional study to analyze the prevalence of androgenic alopecia in postmenopausal women (*n* = 178, age: 50–65 years old) and stated that postmenopausal women had a higher prevalence of androgenic alopecia [11].

## 4. Treatments for Alopecia

Several drugs and treatment strategies were proposed, and some are used to treat hair loss in males and females (Table 1).

Minoxidil was the first drug approved by the FDA to treat hair loss. Minoxidil might prolong the anagen phase, stimulate and elongate hair follicles, and have antiapoptotic effects [15,16]. Studies showed that topical minoxidil (2–5%) treatment increases hair count, hair weight, hair shaft diameter, and anagen: telogen ratio [78]. The use of 5% topical minoxidil for 16 weeks significantly improved the hair count in males [18]. Hypertrichosis, scalp itching, and irritation are some side effects of topical minoxidil treatment. After treatment discontinuation, excessive hair will disappear [78].

Finasteride, an inhibitor of type II 5α reductase, is used to manage and treat male androgenetic alopecia (AGA). Finasteride and 5% topical minoxidil are effective and safe to treat mild to severe AGA; oral finasteride treatment was more efficient [17]. The combination of finasteride and minoxidil treatment significantly improved the hair weight in stump-tail macaque monkeys [79]. Dutasteride is a dual 5α reductase inhibitor and is effective in treating AGA. Dutasteride (2.5 mg/day) treatment was more effective than finasteride (5 mg/day) [80].

Combination therapy is more effective than a single treatment procedure. Generally, treatment for AGA continues for one year, and the initial signs of progress will appear even after four to six months. The regular use of drugs is necessary to achieve their efficiency in hair regrowth. The drug efficiency and hair regrowth vary depending on the subjects and the intensity of the problem.

Topical minoxidil (2%) showed effective hair regrowth in women with low body mass, whereas cyproterone acetate (50 mg) and Diane treatment were effective in women with hyperandrogenism [81]. Finasteride (2.5 mg) treatment effectively induces hair regrowth in pre- and postmenopausal women [82,83,84].

Hair transplantation is the best option for severe AGA in men, and subsequent 1 mg finasteride treatment helps improve surgery results [19]. However, for women, hair transplantation has complications, and some factors affect that. Surgery may induce an effluvium of the pre-existing hair, and hair thinning is spread over the parietal and occipital regions in women.

Cell therapy is effective for hair loss treatment with mild side effects. Thus, the use of stem cells and follicular cells for hair loss treatment is under development. Dermal sheath cup cells (DSCs) have immune tolerance and hair inductivity, which can be isolated from the peribulbar dermal sheath cup. The DSCs-based treatment for hair regrowth required further improvement. Adipose-derived stem cells and dermal papilla cells (DPCs) are the choices for hair growth therapy. Since hair loss is associated with losing DPCs, improved DPCs-based treatment procedures may promote hair growth [20].

People are interested in complementary and alternative medicine, especially using natural products to treat hair loss. Hair loss treatment requires a multisensory approach, and natural products may act as adjuvant therapeutic agents to treat hair loss [21]. EGCG, amino acids, caffeine, capsaicin, curcumin, garlic gel, marine proteins, melatonin, onion juice, procyanidin, pumpkin seed oil, red ginseng extract, rosemary oil, saw palmetto, vitamins, and zinc are reported to have beneficial effects in hair regrowth [21,22].

However, no concrete treatment procedure is currently available to promote permanent hair growth without side effects.

## 5. Role of Phytochemicals in Managing Hair Loss

This section summarizes the representative studies on hair growth-promoting activities of phytochemicals.

The herbal extract was prepared with aqueous extracts of *Urtica dioica* L. root, *Urtica urens* L. leaf, *Equisetum arvense* L. leaf, *Achillea millefolium* L. aerial part, *Matricaria chamomilla* L. flower and *Ceratonia siliqua* L. fruit. The herbal extract mixture (HEM) was rich in vitamins (thiamine, riboflavin, pyridoxine, and ascorbic acid) and flavonoids (myricetin, quercetin, and kaempferol). The treatment of HEM significantly downregulated the expression of IL-1α, one of the important hair loss mediators and growth inhibitory agents, in HaCaT cells compared to the control. The study suggested that HEM could be an adjuvant therapeutic agent for nonscarring alopecia [85].

Cyclic adenosine monophosphate (cAMP), an intracellular messenger, is one of the hair growth-promoting target molecules. Dermal papilla cells (DPCs) are involved in hair morphogenesis and regeneration. The treatment of α-phellandrene supported the proliferation of DPCs and increased the PKA C α (protein kinase A catalytic subunit) and CREB (cAMP-responsive element binding protein). Furthermore, CREB-dependent growth factors, such as VEGF (vascular endothelial growth factor, known for its role in accelerating hair growth) expression, were upregulated in DPCs. The cAMP inhibitors reversed the changes, which showed that α-phellandrene could prevent hair loss via the cAMP-dependent pathway [86].

The ethanolic (EEAS) and aqueous extracts of *Albizia saponaria* (Lour.) Blume ex Miq. barks (AEAS) were studied for their ability to promote hair growth. The EEAS was rich in tannins, alkaloids, flavonoids, saponins, phenols, and triterpenoids. The treatment of EEAS and AEAS, at a concentration of 5–20%, significantly nourished and promoted hair growth in rabbits [87]. León et al. [88] reported that the aqueous extract of latex of *Jatropha gaumeri* Greenm. treatment showed wound-healing effects and hair growth stimulation by facilitating the regeneration of hair follicles in male Balb/c mice [88].

The oral and topical application of β-cyclodextrin inclusion complex containing **γ**-linolenic acid, β-sitosterol, EGCG, and genistein for 270 days showed hair loss preventive effects in androgenetic alopecia male subjects. The baldness in the vertex scalp of subjects was noticed at baseline. After 90 days of treatment, a significant improvement in hair thickening was observed. After 270 days of treatment, there was no evidence of hair loss in the study subjects. The study suggested that a plant-based, nano-complexed formula could be a supportive treatment strategy for androgenetic alopecia and drug-based treatments [89]. Leaves and roots of *Arctium lappa* L. are used to treat skin inflammation, promote wound healing, act against dandruff, and prevent hair loss [90].

## 6. Role of Phytochemicals in Hair Growth

### 6.1. Evidence from In Vitro Studies

*Centella asiatica* L. extracts have polyphenols and flavonoids and exhibit significant antioxidant activity. Particularly, the ethanolic extract of *C. asiatica* induced the expression of VEGF in DPCs, which is responsible for hair growth, indicating that crude extract of *C. asiatica* may be a potent hair growth-promoting candidate [24].

Petroleum ether extract of *Cuscuta reflexa* Roxb. (PECR) prevented the alopecic condition and promoted hair growth in testosterone-treated mice. It promoted hair growth, displayed in the anagen/telogen ratio, follicular hair density, and skin section. The hair growth-promoting activity of PECR was attributed to the 5α-reductase inhibitory activity of the same, i.e., PECR hinders the conversion of testosterone to dihydrotestosterone, thereby preventing the androgen-induced alopecia in mice [25].

*Sterculia urceolata* Sm. leaves extract (SULE) was prepared by the maceration method using ethanol as solvent. Then the extracts were fractionated by liquid–liquid extraction using two or more non-mixed solvents. The SULE contains polyphenols, flavonoids, and terpenoids. The hair growth-stimulating effect of ethanol extract and its fraction were tested in male rabbits. The results showed that the 15% water fraction of ethanolic extract significantly stimulated hair growth [26].

*Centipeda minima* (L.) A. Braun & Asch extract (CMX; contains arnicolide D, arnicolide C, microhelenin C, and brevilin A) promoted hair growth. CMX treatment promoted DPCs proliferation and increased the expression of FZDR (frizzled receptor), Wnt5a (Wnt family member 5a), and VEGF, indicating an increase in the expression of growth factors (VEGF and IGF-1). The phosphorylation of extracellular signal-regulated kinase (ERK) and c-Jun N-terminal kinase (JNK) and accumulation of β- catenin were increased in DPCs. The results suggested that the hair growth-promoting property of CMX is associated with Wnt/ β-catenin (associated with the development of hair follicles and sebaceous glands), ERK, and JNK signaling pathways. The drug-likeness and bioavailability of CMX were appreciable, and CMX could be used to manage hair loss and induce hair growth [91].

The phytochemical composition of the ethanol and petroleum ether extract of *Phyllanthus niruri* L. leaves, *Zingiber officianale* Roscoe rhizomes, and seeds of *Croton tiglium* L. showed that all the samples have saponins. *Phyllanthus niruri* L. and *Zingiber officianale* have phenolic compounds, flavonoids, terpenoids, tannins, alkaloids, and cardiac glycosides. The extract-loaded ethosomal formulations were prepared and studied for hair growth-promoting activity. The number of hair follicles, hair follicular density, and anagen/telogen ratio has increased in the petroleum ether extract of *Croton tiglium*-loaded ethosome and combined petroleum ether extract-loaded ethosome compared to that of the combined ethanol extract-loaded ethosome in keratinocytes. The typical epidermis, hair follicles, and root of hair shift were observed after 21 days of treatment with ethosomal formulations in male Wistar rats. The combined ethanol extracts loaded ethosomal formulations are safe and showed no skin irritation or redness [92].

The growth-promoting property of Phyllotex™ extract with different concentrations of *p*-coumaric acid (herbal formula) was studied. The herbal formula induced the proliferation of DPCs and HaCaT cells and increased the phosphorylation of transcription factors ERK-1 and ERK-2. The herbal formula treatment blocked the TGF-β1 (Transforming growth factor-beta 1) expression without affecting the dihydrotestosterone level and protected the keratinocytes and DPCs from oxidative stress. The study claimed that the herbal formula showed a higher hair growth-promoting activity than the standard drug, minoxidil, and it could be a potent hair growth-promoting agent to treat hair loss [93].

The methanolic extract of *Allium ascalonicum* L. (MEAA) was composed of quercetin, rosmarinic, and *p*-coumaric acids. The MEAA suppressed the lipopolysaccharide (LPS)-induced nitric oxide production in RAW264.7 macrophage cells. MEAA treatment promoted hair growth-promoting activity, attributed to the upregulation of genes related to Wnt/β-catenin, VEGF, and sonic hedgehog (downstream pathway of the Wnt/β-catenin signaling regulates hair follicle induction) pathways in DU-145 cells. The study displayed that MEAA could be a convincing hair growth-promoting agent [94].

The aqueous extract of *Morus alba* L. root (AEMA) promoted hair growth. AEMA induced the anagen phase by activating the β-catenin in DPCs, enhancing the growth factor secretion. The study showed that AEMA has hair growth-promoting potential [95]. AEMA increased the expression of proliferative and anti-apoptotic genes in human dermal fibroblasts and promoted the expression of hair-growth-related genes in DPCs. AEMA-treated human dermal fibroblasts also secreted the angiogenic paracrine factors. The results indicated that AEMA could prevent hair loss [96].

### 6.2. Evidence from In Vivo Studies

Inflammation is one of the key events associated with the etiology of alopecia. Thus, herbs with anti-inflammatory and immunomodulating properties might be promising bioactive candidates to manage the severity of alopecia. The herb preparation consists of *Arctium lappa* roots, *Sophora japonica* L. fruits, *Acorus calamus* L. roots, *Urtica dioica* leaves, *Humulus lupulus* L. fruits (rich in terpenoids, flavonoids, amino acids, and polyphenolic compounds) showed moderate anti-inflammatory and immunomodulating activity in mice model [97].

The natural plant extract (NPE) consists of a mixture of cnidium (*Ligusticum chuanxiong* Hort), Korean angelica (*Angelica gigas* Nakai) root, licorice (*Glycyrrhiza uralensis* Fisch. ex DC.) root, Morus bark (*Morus alba* L.), pine (*Pinus densiflora* Siebold & Zucc.) needle, sophora (*Sophora angustifolia* Siebold & Zucc.) root, and sweet flag (*Acorus calamus* L.) (80% asarone, 18% cumaric acid and 2% of others) in a specified ratio (NJY Biotechnology Co., Ltd., Seoul, Republic of Korea) were reported for its hair loss prevention and hair growth promotion property in C57BL/6 mice. The hair thickness, density, and length were significantly higher in NPE treated group compared to the control. NPE increased the expression of KEF (Keratinocyte growth factor) and VEGF while down-regulating the TGF-β1. The results suggested that NPE could prevent hair loss and promote growth [98].

The hair growth-promoting property of Noni (*Morinda citrifolia* L.)-fruit extracts and their sub-fractions (rich in alkaloids, flavonoids, phytosterols, anthraquinones, saponins, and tannins) was reported using the DHT-induced alopecia rabbit model. Furthermore, in silico study revealed that three alkaloid compounds with skeleton piperazine and piperidine exhibited a similar binding affinity to minoxidil. The androgen receptor compounds exhibited acceptable stability regarding root-mean-square deviation and solvent-accessible surface values. The study results revealed that some of the alkaloids of Noni fruit have anti-alopecia-like properties [99].

Joshi et al. reported that the mixture of aqueous fractions of *Ficus religiosa* L. and *M. alba* L. leaf extract (topical application for 28 days) promoted hair growth and hair follicles regeneration by inducing the anagen phase in BALB/c athymic nude mice [100].

The plant extract containing *Acorus calamus* var. *angustatus* promoted hair growth in C57BL/6 mice. The hair thickness, density, and length were significantly higher in the plant extract-treated group compared to the 5% minoxidil-treated and control groups. The histological examination showed that the hair follicles and shaft were healthy. The result claimed that plant extract could promote hair growth and maintain the health of hair roots [101]. *β-asarone* isolated from the *Acorus calamus* root showed moderate hair growth-promoting activity in Wistar rats [102].

Rusu et al. [103] conducted a preclinical study using rabbits to analyze the hair growth and hair regeneration effect of castor oil (*Ricinus communis* L.) lotion (lotion containing 35% or 40 % castor oil). The lotion containing 35% of castor oil treatment (topical application of lotion for 1 month) increased the length, softness, and thickness of the hair in more than 50% of the animals compared to the blank area (area not treated with castor oil lotion) and showed no adverse effects. The hair growth upon 35% castor oil lotion treatment in healthy rabbits indicated the hair regeneration effect of castor oil [103].

Kporou et al. [104] investigated the hair growth activity, safety, and quality of an ointment containing castor oil (*Ricinus communis*) as an active ingredient and shea butter from *Butyrospermum parkii* (G.Don) Kotschy (excipient) using rabbits. The topical application of the ointment on the shaved areas of the rabbits for 28 days showed hair growth and increased hair mass, indicating the growth-promoting ability of castor oil [104].

The extract was obtained by boiling the *Citrus sinensis* (L.) Osbeck (pro. sp.) peel (70%), *Linum usitatissimum* L. seeds (5%), *Nigella sativa* L. (8%), *Trigonella foenum-graecum* L. (10%), and *Zingiber officinale* Roscoe roots (5%) in water (500 mL) for 15 min and allowed to cool. The hair serum was prepared by adding castor oil (0.5%) and vitamin E (1.5%) to the filtered extract. The topical application of hair serum showed hair growth and increased the hair weight of the treated rabbits [105].

The methanolic extract of *Eclipta alba* (L.) Hassk, *Asiasari radix*, and *Panax ginseng* C. A. Mey were dried and mixed in vehicle solution (propylene glycol: ethanol: dimethyl sulfoxide at the ratio of 67: 30: 3% *v*/*v*). Its hair growth-promoting effect was studied in nude (Foxn1^nu^) mice. The preparations, rich in wedelolactone, were topically applied on the back of mice every day until the completion of 2 full hair growth cycles. *E. alba* extract-treated mice showed significant hair density and distinct hair growth area compared to the control. Further, *E. alba* extract treatment has increased the number of hair follicles, and it has been confirmed that the presence of follicular keratinocytes and cells in the S-phase of the cell cycle. Altogether, the results indicated that *E. alba* extract has potent hair growth-promoting properties, which could be used to develop complementary treatments for hair loss [106].

The ethanolic extract of *Carthamus tinctorius* L. florets-loaded nanostructured lipid carrier (CTNLC) was prepared, and its hair growth-promoting property was tested in male C56BL/6Mlac mice. The topical application of CTNLC for 28 days promoted hair growth in mice, and its efficiency was better than the minoxidil [107]. Petroleum ether and ethanolic extracts of *Trigonella foenum-graecum* leaves showed hair growth-promoting activity in mice. The extracts are rich in polyphenols, flavonoids, polysaccharides, and glyco-saponins. The topical application of extracts for 21 days improved hair length and diameter [108]. The methanol (95%) extract of *Carthamus caeruleus* L. roots (MECC) was rich in tannins, anthocyanins, flavonoids, senosids, quinones, glucosides, mucilages, coumarins, and saponosides. MECC exhibited anti-inflammatory (reduced edema), wound healing, and hair growth-promoting activities in vivo models. Application of MECC-ointment showed significant hair growth compared to control in Wister rats [109].

The powdered rhizome of *Zingiber officinale* was extracted using different solvents such as water, alcohol, and alcohol: water (1:1). The extracts have alkaloids, flavonoids, and tannins. The herbal gels were prepared with 5% extract and carbopol 934 (served as the gel base) and studied for hair growth-stimulating activity. The hydroalcoholic extract containing herbal gel exhibited high spreadability, homogeneity, viscosity, and pH. The gel showed negligible skin irritation and was stable at different temperatures. The hydroalcoholic extract containing herbal gel showed significant hair growth-promoting properties comparable to minoxidil (28 vs. 25 mm of hair length) [110].

Sakib et al. [111] reported the hair growth-promoting property of the *n*-hexane fraction of the methanol extract of *Leea indica* (Burm. F.) Merr. Leaves (HMLIL) in Swiss Albino mice. Phthalic acid, farnesol, palmitic acid, *n*-tricosane, *n*-tetracosane, and *n*-heptacosane are abundant in the HMLIL. Different concentrations of HMLIL (0.1, 1, and 10%) and minoxidil were topically applied on the shaved dorsal portion of mice for 21 days. The animals treated with HMLIL showed significant hair growth compared to the control. Especially, 1% of HMLIL showed high hair growth than other tested concentrations. The study also revealed the hemolysis inhibitory property and thrombolytic activity of HMLIL. In silico analysis revealed that Phthalic acid, farnesol, *n*-tricosane, *n*-tetracosane, and *n*-heptacosane present in the HMLIL have the ligand binding ability towards PGD2 synthase (one of the crucial plays involved in the hair loss). The study claimed that *Leea indica* could be a potent alternative to manage hair loss [111].

The pTOPFLASH DNA construct with T cell factor (TCF)-binding sequence for the β-catenin TCF complex was employed to measure the activation of Wnt/β-catenin signaling. Wnt/β-catenin signaling is involved in hair growth promotion in mice and DPCs. The molecular docking showed that tectoridin (an active compound present in the ethanolic extract of the rhizome of *Belamcanda chinensis* (L.) DC. (ERBC)) could stimulate the promoter construct, inducing the transcription of pTOPFLASH in the transfected fibroblasts. The hair growth-promoting property of tectoridin was demonstrated in DPCs and mouse vibrissae organ culture. Tectoridin treatment activates the Wnt signaling in DPCs, which was confirmed through TCF/LEF1 (Lymphoid enhancer-binding factor-1) transcriptional activity, expression of AXIN2 (Axin-related protein), β-catenin, LEF-1, IGF-1 (Insulin-like growth factor 1), and alkaline phosphatase. The hair shaft extension was reported in tectoridin-treated cultured mouse vibrissae. The study stated that ERBC and tectoridin have hair growth-promoting abilities [27].

### 6.3. Evidence from Clinical Trials

Scalp shampoo containing 0.1% *Caesalpinia sappan* L. bark and 0.3% *Inula helenium* L. extract (SCSIH) has been reported for its hair growth-promoting properties in healthy humans with androgenetic alopecia. The volunteers were asked to use 3 mL of shampoo twice daily for 24 weeks, and the changes (hair density and hair count) were observed at baseline and after 8, 16, and 24 weeks using a phototrichogram. The hair count was significantly increased in the shampoo-treated group after 16 and 24 weeks compared to baseline and controls. The results showed that the SCSIH could promote hair growth and prevent hair loss [112].

Ablon [113] investigated a randomized, double-blind, placebo-controlled study and evaluated the effect of oral supplementation (for six months) of a commercial product (Viviscal^®^ Professional Strength; one tablet twice a day) in healthy women having self-perceived thinning hair due to stress, poor intake of food, hormonal imbalance, or due to the menstrual cycles. Viviscal^®^ Professional Strength product contains AminoMar C™ marine complex (a blend of shark and mollusk powder), silica derived from *Equisetum* sp. (horsetail), vitamin C derived from *Malpighia emarginata* Sessé & Moc. Ex DC., procyanidin B-2, L-cystine, and L-methionine. Daily intake of Viviscal^®^ Professional Strength supplement increased the terminal hair count and terminal hair diameter after 90 and 180 days of intervention compared to the baseline and that of the placebo group. In contrast, the supplement increased the vellus hair count compared to the baseline after 180 days of intervention. The twice-daily administration of new Viviscal Professional Strength oral tablets significantly increased the number of terminal and vellus hairs. It increased hair diameter in women with self-perceived thinning hair compared to baseline values in this group and those taking the placebo. Most study participants believed that using the new oral supplement significantly improved skin and hair quality and quality of life [113].

Ablon and Kogan investigated the effect of a nutraceutical supplement (Nutrafol^®^ women’s capsules). Nutrafol^®^ women’s capsules contain phytoactive extracts, phytochemicals, vitamins, and minerals. The hair growth-promoting property of Nutrafol^®^ has been studied in healthy perimenopausal, menopausal, and postmenopausal women. Six months of oral supplementation of Nutrafol^®^ significantly increased the terminal and total hair count compared to the placebo. The hair shedding also significantly reduced (about 32.41% after 6 months) compared to the placebo. The study showed that Nutrafol^®^ was safe and promoted hair growth [114]. Similarly, Nutrafol^®^ supplementation significantly improved the hair growth rate, thickness, growth, and volume in women with self-perceived thinning hair after six months compared to the placebo [115].

The emulsion extract of brevilin A from *Centipeda minima* (BA-CMX) inhibited JAK3 (Janus Kinase 3) in a dose-dependent way. Healthy subjects with mild to moderate vertex balding were treated with BA-CMX (1% brevilin A) once daily for 24 weeks. The total hair count, anagen, and terminal hair count were increased compared to the baseline values after 24 weeks. The systems pharmacology approach showed that BA-CMX targets JAK-STAT and MAPK pathways and cytokine–cytokine receptor interactions. This study claimed that BA-CMX could be useful in treating mild to moderate vertex balding [116].

The application of NPE for ten mon”hs s’gnificantly prevented hair loss in alopecia subjects. Furthermore, the thickness and density of the hair were increased compared to baseline values. The study claimed natural plant extract could effectively prevent hair loss [117].

Apart from the studies mentioned above, several in vitro, in vivo, and preclinical studies were conducted on hair growth-promoting properties of epigallocatechin gallate (EGCG) [118], caffeine [119], capsaicin [120], curcumin, garlic gel [22], marine proteins [121], melatonin [22], onion juice [122], procyanidin [123], pumpkin seed oil [124], Red ginseng extract [125], rosemary oil, saw palmetto [126], and vitamins [21]. The findings were recently reviewed and published [22] (Table 2).

## 7. Mechanisms Associated with Hair Loss Prevention/Hair Growth-Stimulating Property of Phytochemicals

**Figure 2:**
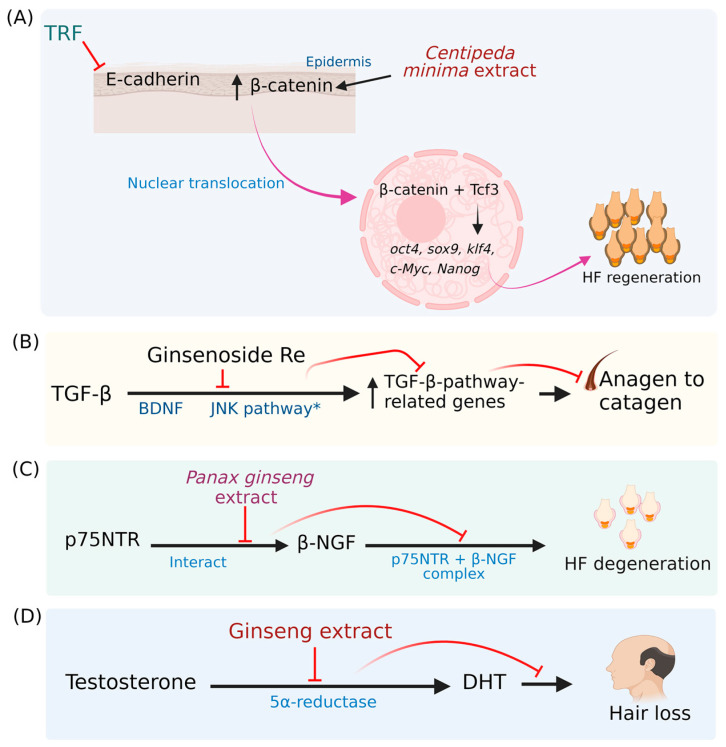
The possible mechanisms behind the hair growth-promoting properties of phytochemicals. (**A**) TRF inhibits the epidermal E-cadherin and facilitates the translocation of β-catenin into the nucleus. β-catenin combined with Tcf3 and β-catenin-Tcf3 decoy may induce the *oct4*, *sox9*, *klf4*, *c-Myc*, and *Nanog*, resulting in hair follicular regeneration. *Centipeda minima* extract could also activate the β-catenin and promotes hair follicular regeneration. (**B**) TGF-β pathway-related genes could be upregulated by BDNF and activated JNK pathway, which promotes anagen to catagen. Ginsenoside Re inhibits the TGF-β pathway-related gene activation and prevents the catagen stage. (**C**) The formation of p75NTR and β-NGF complex accelerates hair follicle degeneration. *Panax ginseng* (*P. ginseng*) extract could inhibit the complex formation. (**D**) The conversion of testosterone to DHT induces hair loss. The inhibition of 5αR prevents the formation of DHT and hair loss. Ginseng extract showed potent 5αR inhibitory activity [28,91,135,136,137,138]. TRF: Tocotrienol-rich fraction; Tcf3: Transcription factor 3; *oct4*: Octamer-binding transcription factor 4; *sox9*: SRY-Box transcription factor 9; *klf4*: Kruppel-like transcription factor 4; *c-Myc*: Proto-oncogene transcription factor; *Nanog*: Nanog Homeobox transcription factor 4; TGF-β: Transforming growth factor-beta; BDNF: Brain-derived neurotrophic factor; JNK: c-Jun-N-terminal kinase; p75NTR: p75 neurotrophin receptor; β-NGF: β-nerve growth factor; DHT: 5α-dihydrotestosterone; 5αR: 5α-reductase. * Hyperactivation of the JNK pathway (The figure was created using BioRender.com; accessed on 17 November 2022).

**Figure 3:**
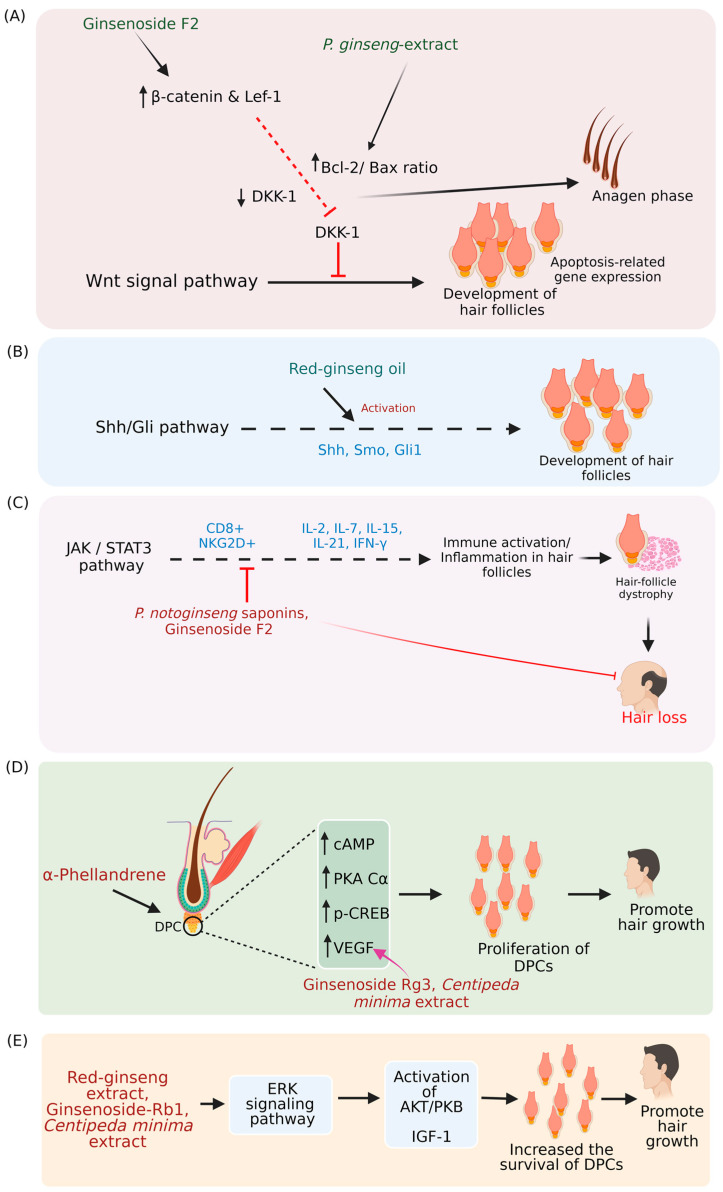
The possible mechanisms behind the hair growth-promoting properties of phytochemicals. (**A**) Wnt signaling promotes hair follicles development, and DKK-1 could hinder Wnt signaling. Ginsenoside F2 induces the formation of the β-catenin-Lef-1 complex, suppressing the DKK-1 expression, thereby promoting hair follicle development. *P. ginseng* extract increases the Bcl-2/Bax ratio, which stimulates the anagen phase. (**B**) Activation of the Shh/Gli pathway induces hair follicles development, and red ginseng oil activates the Shh/Gli pathway. (**C**) The activation of the JAK/STAT3 pathway causes hair follicle dystrophy through inflammation. *Panax notoginseng* (*P. notoginseng*) saponins and ginsenoside F2 suppress immune activation and prevents hair loss. (**D**) α-phellandrene promotes DPCs proliferation by increasing the c-AMP, PKA Cα, CREB, and VEGF and promotes hair growth. *Centipeda minima* extract, and ginsenoside Rg3 could induce VEGF expression and facilitate hair growth. (**E**) ERK signaling, AKT/PKB action, and IGF-1 increase the survival of DPCs, thereby promoting hair growth. *Centipeda minima* extract and ginsenoside Rb-1, red ginseng extract activates the pathway [29,86,139,140,141,142,143]. Wnt: Wingless-type MMTV integration site family member; DKK-1: Dickkopf WNT signaling pathway inhibitor 1: Lef-1: Lymphoid enhancer-binding factor 1; Bcl-2: B-cell lymphoma 2; Bax: Bcl-2-associated X; Shh: Sonic hedgehog; Gli: Glioma-associated oncogene homolog; JAK: Janus-activated kinase; STAT3: Signal transducer and activator of transcription 3; DPCs: Dermal papillary cells; c-AMP: Cyclic adenosine monophosphate; PKA Cα: Protein kinase A catalytic subunit α; CREB: cAMP-responsive element binding protein; VEGF: Vascular endothelial growth factor; ERK: Extracellular-signal-regulated kinases; AKT(Serine/threonine kinase)/PKB: Protein kinase B; IGF-1: Insulin-like growth factor 1. (The figure was created using BioRender.com; accessed on 17 November 2022).

Diverse mechanisms of hair growth-promoting activity of phytochemicals have been proposed and demonstrated. TGF-β (Transforming growth factor-beta), JNK (c-Jun-N-terminal kinase), JAK (Janus-activated kinase)/STAT3 (Signal transducer and activator of transcription-3), WNT (Wingless-type MMTV integration site family member) WNT/β-catenin, BMP (Bone morphogenetic proteins)/Smad (homologs of the Drosophila protein, mothers against decapentaplegic (Mad) and the *Caenorhabditis elegans* protein Sma) and Shh (Sonic hedgehog)/Gli (Glioma-associated oncogene homolog) pathways were involved in the hair cycle and growth [24,25,26,27,28,29,30] (Figure 2 and Figure 3).

Tocotrienol-rich fraction silenced epidermal E-cadherin and translocated the β-catenin. β-catenin interacts with Tcf3 (transcription factor 3), and β-catenin-Tcf3 decoy induces the *oct4* (Octamer-binding transcription factor 4), *sox9* (SRY-Box transcription factor 9), *klf4* (Kruppel-like transcription factor 4), *c-Myc* (proto-oncogene transcription factor) and *Nanog* (Nanog Homeobox transcription factor 4), which induces the hair follicular regeneration. The results showed that the downregulation of E-cadherin activates β-catenin [28].

The hair growth-promoting properties of ginseng extracts are attributed to several molecular pathways [29]. The activation of the TGF-β-pathway promoted the catagen phase of the hair cycle and was also associated with the activated JNK pathway. Ginsenoside’s downregulation of TGF-β pathway-related genes promotes hair growth [135,136].

The interaction of neurotrophin and its receptors is necessary to initiate its function, and neurotrophin receptor antagonists prevent the further molecular reaction. p75NTR (p75 neurotrophin receptor) and β-NGF (β-nerve growth factor) interaction influences hair follicles degeneration. *P. ginseng* extract inhibited the interaction of p75NTR and β-NGF and could promote hair growth [137].

Exposure to androgen causes hair loss. The conversion of testosterone to DHT (5α-dihydrotestosterone) is facilitated by the 5αR (5α-reductase) enzyme in hair follicles. The inhibition of 5αR prevents hair loss. Ginsenoside Rg3 and Rd showed 5αR inhibitory activity and prevented hair growth suppression in mice [140].

WNT signaling has a role in the development of hair follicles. The suppression of WNT signaling by DKK1 (Dickkopf WNT signaling pathway inhibitor 1) could prevent hair follicle formation [144]. Ginsenoside F2 induced the β -catenin and Lef-1 (Lymphoid enhancer-binding factor 1) and suppressed the DKK1 in DPCs, which further improved the Bcl-2 (B-cell lymphoma 2)/Bax (Bcl-2-associated X) ratio, revealed that ginseng extract could suppress the DKK1 mediated activation of catagen, and regulates the apoptosis-associated gene expression in hair follicles [139,140].

Shh/Gli is involved in hair follicle growth and cycle, and its importance has been demonstrated in mice models [29]. Red ginseng oil improved hair growth via Shh/Gli pathway in testosterone-treated mice [141].

Hair follicles are immune-tolerated cells. Losing immune tolerance causes hair follicle dystrophy and promotes the catagen phase [145]. JAK/STAT3 pathway activates the immune cells, so the inhibition of JAK/STAT3 might promote hair follicle regeneration [146]. It has been reported that the ginsenosides affect the JAK2 signaling and suppress NKG2D+ T (NK group 2D-positive T cells) cells. Furthermore, the inhibition of IL-17A (Interleukin-17a) promoted hair growth [147]. *P. notoginseng* saponins and ginsenoside F2 weakened the Th17 (T helper 17) and IL-17A activity and promoted hair growth [142,143].

Ginsenosides promoted hair growth by activating VEGF (Vascular endothelial growth factor) and CD34 (Cluster of differentiation 34). It has been reported that the red ginseng extract and ginsenoside-Rb1 activated the Bcl-2, ERK, and AKT (Serine/threonine kinase)/PKB (Protein kinase B) pathways, which promotes the proliferation of DPCs [29].

Recently, Kang et al. [86] described that the α-phellandrene promoted DPCs proliferation by increasing the c-AMP (Cyclic adenosine monophosphate), PKA Cα (protein kinase A catalytic subunit α), and CREB (cAMP-responsive element binding protein). Specifically, α-phellandrene upregulated the VEGF in DPCs [86]. Similarly, *Centella asiatica* L. extract confers the hair growth-promoting activity through the induced expression of VEGF in DCPs [24], and ginsenoside rg3 up-regulates the VEGF in DCPs and mouse hair follicles [138].

Kim et al. [91] showed that the hair growth-promoting property of CMX is attributed to the activation of VEGF, WNT/β-catenin, ERK (Extracellular-signal-regulated kinase), and JNK signaling pathway. CMX upregulated the expression of VEGF, Wnt5a (Wnt family member 5a), and FZDR (Frizzled receptor), which enhanced the IGF-1 (Insulin-like growth factor 1) expression in DPCs. Subsequently, the β-catenin level increased, facilitating the activation of ERK, JNK, and GSK (Glycogen synthase kinase-3), promoting the regeneration of DPCs [91].

Apart from the discussed mechanisms, the hair growth-promoting effect of watercress oil was attributed to the activation of MAPK1 (Mitogen-activated protein kinase 1), MAPK3 (Mitogen-activated protein kinase 3), and MAPKp38 (p38 mitogen-activated protein kinases) receptors [148].

## 8. Conclusions

The literature survey revealed that phytochemicals have beneficial effects on hair health. The underlying mechanisms associated with hair growth-promoting properties of the phytochemicals are detailed. The results showed that hair growth-promoting properties of phytochemicals are associated with the TGF-β pathway, β-catenin, JAK/STAT3 pathway, ERK signaling pathway, and 5α-reductase inhibitory property. Some studies proposed that phytochemicals are more effective than conventional hair loss regimens such as minoxidil and finasteride. However, further studies are recommended to refine the findings. The current review provided updated information on the role of phytochemicals in hair health and associated mechanisms, which may aid in developing phytocompound-based therapeutics to manage hair problems.

## Figures and Tables

**Table 1:** The common treatment for hair loss [22].

Hair Loss Type	Treatments
Androgenic alopecia	Topical minoxidil (2 to 5%), oral finasteride/dutasteride, hair transplantation
Alopecia areata	Topical minoxidil (5%), corticosteroids (Topical or oral)
Alopecia totalis	Hair transplantation
Cicatricial alopecia	Topical or oral antibiotics, corticosteroids, scalp reduction surgery, hair transplantation
Senescent alopecia	Topical minoxidil
Traction alopecia	Anti-inflammatory agents, hair transplantation
Telogen effluvium	5-α reductase inhibitors

**Table 2:** The representative phytochemicals with hair growth-promoting properties [20].

Phytochemicals	Mechanism of Action	References
Caffeine	5α-reductase inhibition. Stimulates the HFK and ORS proliferation. Reduces oxidative stress. Reduces apoptosis and necrosis.	[119,127,128]
Epigallocatechin gallate (*Camellia sinensis* (L.) Kuntze)	Extension of analgen phase. Reduces oxidative cell damage and oxidative stress. Reduces the senescence-related gene expression. Inhibits the IFN-γ signaling.	[118,129,130]
Procyanidin B2	Suppresses inflammation.	[123]
Herbal mix * (*Chamaemelum nobile* (L.) All., *Althaea officinalis* L., *Persea Americana* Mill., *Rosmarinus officinalis* L., *Aloe vera* (L.) Burm. F., *Urtica dioica, Thymus vulgaris* L.)	5α-reductase 2 inhibition. Inhibits apoptosis.	[131]
Herbal mix (*Urtica uren* L., *Urtica dioica, Matricaria chamomilla*, *Achillea millefolium*, *Ceratonia siliqua*, *Equisetum arvense*) contains vitamins B_1_, B_2_, B_6_ and C, and myricetin, quercetin, kaempferol, and trace elements (iron, zinc, and copper)	Induces the HF effects.	[132]
Vitamin C, zinc, horsetail stem extract, and flax seed extract *	Systemic effects.	[121]
Capsaicin (*Capsicum annuum* L.)	Stimulates the perifollicular nerves. Stimulates the HF immune system.	[120]
Crude onion juice (*Allium cepa* L.) *	Induction of immunological reaction and antigenic competition.	[122]
Red ginseng extract (*Panax ginseng* C.A. Mey.)	Increased cell proliferation.	[125]
Red ginseng extract + ginsenoside Rb1 and ginsenoside Rg3	Induced upregulation of androgen receptor.	[125]
Pumpkin seed oil *	5α-reductase inhibition.	[124]
Saw palmetto extract *	5α-reductase inhibition.	[133,134]

* Phytocompounds in the plant extracts were not detailed in the study.

## Data Availability

Data sharing not applicable.

## References

[1] Broadley D., McElwee K.J. (2020). A “hair-raising” history of alopecia areata. Exp. Dermatol..

[2] Park A.M., Khan S., Rawnsley J. (2018). Hair biology: Growth and pigmentation. Facial Plast. Surg. Clin. N. Am..

[3] Mulinari-Brenner F., Neto F.J., Rosas F.M.B., Torres L.F.B. (2006). Morphometry of normal scalp hair follicles. An. Bras. Dermatol..

[4] Wosicka H., Cal K. (2010). Targeting to the hair follicles: Current status and potential. J. Dermatol. Sci..

[5] Trüeb R.M. (2005). Aging of hair. J. Cosmet. Dermatol..

[6] Trüeb R.M., Henry J.P., Davis M.G., Schwartz J.R. (2018). Scalp condition impacts hair growth and retention via oxidative stress. Int. J. Trichol..

[7] Yu V., Juhász M., Chiang A., Atanaskova Mesinkovska N. (2018). Alopecia and associated toxic agents: A systematic review. Skin Appendage Disord..

[8] Mercke Y., Sheng H., Khan T., Lippmann S. (2000). Hair loss in psychopharmacology. Ann. Clin. Psychiatry.

[9] Chumlea W.C., Rhodes T., Girman C.J., Johnson-Levonas A., Lilly F.R., Wu R., Guo S.S. (2004). Family history and risk of hair loss. Dermatology.

[10] Łukasik A., Kozicka K., Kłosowicz A., Jaworek A., Wojas-Pelc A. (2021). The role of family history and its influence on the onset time in female pattern hair loss. Postepy Dermatol. Alergol..

[11] Choi S., Zhang B., Ma S., Gonzalez-Celeiro M., Stein D., Jin X., Kim S.T., Kang Y.L., Besnard A., Rezza A. (2021). Corticosterone inhibits GAS6 to govern hair follicle stem-cell quiescence. Nature.

[12] Su L.H., Chen T.H. (2007). Association of androgenetic alopecia with smoking and its prevalence among Asian men: A community-based survey. Arch. Dermatol..

[13] Chaikittisilpa S., Rattanasirisin N., Panchaprateep R., Orprayoon N., Phutrakul P., Suwan A., Jaisamrarn U. (2022). Prevalence of female pattern hair loss in postmenopausal women: A cross-sectional study. Menopause.

[14] Guo E.L., Katta R. (2017). Diet and hair loss: Effects of nutrient deficiency and supplement use. Dermatol. Pract. Concept..

[15] Messenger A.G., Rundegren J. (2004). Minoxidil: Mechanisms of action on hair growth. Br. J. Dermatol..

[16] Rogers N.E., Avram M.R. (2008). Medical treatments for male and female pattern hair loss. J. Am. Acad. Dermatol..

[17] Arca E., Açikgöz G., Taştan H.B., Köse O., Kurumlu Z. (2004). An open, randomized, comparative study of oral finasteride and 5% topical minoxidil in male androgenetic alopecia. Dermatology.

[18] Olsen E.A., Whiting D., Bergfeld W., Miller J., Hordinsky M., Wanser R., Zhang P., Kohut B. (2007). A multicenter, randomized, placebo-controlled, double-blind clinical trial of a novel formulation of 5% minoxidil topical foam versus placebo in the treatment of androgenetic alopecia in men. J. Am. Acad. Dermatol..

[19] Leavitt M., Perez-Meza D., Rao N.A., Barusco M., Kaufman K.D., Ziering C. (2005). Effects of finasteride (1 mg) on hair transplant. Dermatol. Surg..

[20] Sung J.H. (2023). Effective and economical cell therapy for hair regeneration. Biomed. Pharmacother..

[21] Hosking A.M., Juhasz M., Atanaskova Mesinkovska N. (2019). Complementary and alternative treatments for alopecia: A comprehensive review. Skin Appendage Disord..

[22] Daniels G., Akram S., Westgate G.E., Tamburic S. (2019). Can plant-derived phytochemicals provide symptom relief for hair loss? A critical review. Int. J. Cosmet. Sci..

[23] Padule K., Shinde S., Chitlange S., Giram P., Nagore D. (2022). The advancement of herbal-based nanomedicine for hair. Cosmetics.

[24] Saansoomchai P., Limmongkon A., Surangkul D., Chewonarin T., Srikummool M. (2018). Enhanced VEGF expression in hair follicle dermal papilla cells by *Centella asiatica* Linn. Chiang Mai Univ. J. Nat. Sci..

[25] Pandit S., Chauhan N.S., Dixit V.K. (2008). Effect of *Cuscuta reflexa* Roxb on androgen-induced alopecia. J. Cosmet. Dermatol..

[26] Mustarichie R., Wicaksono I.A. (2019). Hair growth stimulants activity from *Sterculia urceolata* JE Smith ethanol extract. Res. J. Pharm. Technol..

[27] Yuen G.K., Ho B.S., Lin L.S., Dong T.T., Tsim K.W. (2022). Tectoridin stimulates the activity of human dermal papilla cells and promotes hair shaft elongation in mouse vibrissae hair follicle culture. Molecules.

[28] Ahmed N.S., Ghatak S., El Masry M.S., Gnyawali S.C., Roy S., Amer M., Everts H., Sen C.K., Khanna S. (2017). Epidermal E-cadherin dependent β-catenin pathway is phytochemical inducible and accelerates anagen hair cycling. Mol. Ther..

[29] Choi B.Y. (2018). Hair-growth potential of ginseng and its major metabolites: A review on its molecular mechanisms. Int. J. Mol. Sci..

[30] Lin W.H., Xiang L.J., Shi H.X., Zhang J., Jiang L.P., Cai P.T., Lin Z.L., Lin B.B., Huang Y., Zhang H.L. (2015). Fibroblast growth factors stimulate hair growth through β-catenin and Shh expression in C57BL/6 mice. BioMed Res. Int..

[31] Coleman E. (2020). Types and Treatment of Hair Loss in Men and Women. Plast. Surg. Nurs..

[32] Asghar F., Shamim N., Farooque U., Sheikh H., Aqeel R. (2020). Telogen effluvium: A review of the literature. Cureus.

[33] Harrison S. (2002). Sinclair, R. Telogen effluvium. Clin. Exp. Dermatol..

[34] Nnoruka E.N., Obiagboso I., Maduechesi C. (2007). Hair loss in children in South-East Nigeria: Common and uncommon cases. Int. J. Dermatol..

[35] Headington J. (1993). Telogen effluvium. new concepts and review. Arch. Dermatol..

[36] Messenger A.G., McKillop J., Farrant P., McDonagh A.J., Sladden M. (2012). British Association of Dermatologists’ guidelines for the management of alopecia areata 2012. Br. J. Dermatol..

[37] Lundin M., Chawa S., Sachdev A., Bhanusali D., Seiffert-Sinha K., Sinha A.A. (2014). Gender differences in alopecia areata. J. Drugs Dermatol..

[38] Filbrandt R., Rufaut N., Jones L., Sinclair R. (2013). Primary cicatricial alopecia: Diagnosis and treatment. CMAJ.

[39] Villablanca S., Fischer C., García-García S.C., Mascaró-Galy J.M., Ferrando J. (2017). Primary scarring alopecia: Clinical-pathological review of 72 cases and review of the literature. Skin Appendage Disord..

[40] Kyei A., Bergfeld W.F., Piliang M., Summers P. (2011). Medical and environmental risk factors for the development of central centrifugal cicatricial alopecia. Arch. Dermatol..

[41] Finner A.M. (2013). Nutrition and hair. Deficiencies and supplements. Dermatol. Clin..

[42] MacFarquhar J.K., Broussard D.L., Melstrom P., Hutchinson R., Wolkin A., Martin C., Burk R.F., Dunn J.R., Green A.L., Hammond R. (2010). Acute selenium toxicity associated with a dietary supplement. Arch. Intern. Med..

[43] Hammel C., Kyriakopoulos A., Behne D., Gawlik D., Brätter P. (1996). Protein-bound selenium in the seeds of coco de mono (*Lecythis ollaria*). J. Trace. Elem. Med. Biol..

[44] Müller D., Desel H. (2010). Acute selenium poisoning by paradise nuts (*Lecythis ollaria*). Hum. Exp. Toxicol..

[45] Morinaga H., Mohri Y., Grachtchouk M., Asakawa K., Matsumura H., Oshima M., Takayama N., Kato T., Nishimori Y., Sorimachi Y. (2021). Obesity accelerates hair thinning by stem cell-centric converging mechanisms. Nature.

[46] James M.J., Gibson R.A., Cleland L.G. (2000). Dietary polyunsaturated fatty acids and inflammatory mediator production. Am. J. Clin. Nutr..

[47] Goluch-Koniuszy Z.S. (2016). Nutrition of women with hair loss problem during the period of menopause. Menopause Rev..

[48] Lee W.S., Ro B.I., Hong S.P., Bak H., Sim W.Y., Kim D.W., Park J.K., Ihm C.W., Eun H.C., Kwon O.S. (2007). A new classification of pattern hair loss that is universal for men and women: Basic and specific (BASP) classification. J. Am. Acad. Dermatol..

[49] Flores A., Schell J., Krall A.S., Jelinek D., Miranda M., Grigorian M., Braas D., White A.C., Zhou J.L., Graham N.A. (2017). Lactate dehydrogenase activity drives hair follicle stem cell activation. Nat. Cell Biol..

[50] Figlak K., Paus R., Williams G., Philpott M. (2019). 597 Outer root sheath is able to synthesise glycogen from lactate-investigating glycogen metabolism in human hair follicles. J. Investig. Dermatol..

[51] Shi X., Tuan H., Na X., Yang H., Yang Y., Zhang Y., Xi M., Tan Y., Yang C., Zhang J. (2023). The association between sugar-sweetened beverages and male pattern hair loss in young men. Nutrients.

[52] Wu M.L., Deng J.F., Lin K.P., Tsai W.J. (2013). Lead, mercury, and arsenic poisoning due to topical use of traditional Chinese medicines. Am. J. Med..

[53] Namba Y., Suzuki R., Sasaki J., Takayasu M., Watanabe K., Kenji D., Hayashi M., Kitamura Y., Kawamo M., Masaki H. (2013). Thallium group poisoning incident in Japan 2011. Crit. Care.

[54] Sharquie K.E., Ibrahim G.A., Noaimi A.A., Hamudy H.K. (2011). Outbreak of thallium poisoning among Iraqi patients. JSSDDS.

[55] Moeschlin S. (1980). Thallium poisoning. Clin. Toxicol..

[56] Fernando R., Fernando D.N. (1990). Poisoning with plants and mushrooms in Sri Lanka: A retrospective hospital-based study. Vet. Hum. Toxicol..

[57] Nakamura N., Fujita T., Murakami R., Kumasaka R., Shimada M., Shimaya Y., Osawa H., Yamabe H., Okumura K., Yachie A. (2012). A case of familial Mediterranean fever-associated systemic amyloidosis. CEN Case Rep..

[58] Alpsoy E., Leccese P., Emmi G., Ohno S. (2021). Treatment of Behçet’s disease: An algorithmic multidisciplinary approach. Front. Med..

[59] Richette P., Bardin T. (2010). Colchicine for the treatment of gout. Expert Opin. Pharmacother..

[60] Finkelstein Y., Aks S.E., Hutson J.R., Juurlink D.N., Nguyen P., Dubnov-Raz G., Pollak U., Koren G., Bentur Y. (2010). Colchicine poisoning: The dark side of an ancient drug. Clin. Toxicol..

[61] Folpini A., Furfori P. (1995). Colchicine toxicity-clinical features and treatment. Massive overdose case report. J. Toxicol. Clin. Toxicol..

[62] Levsky M.E., Miller M.A., Masneri D.A., Borys D. (2008). Colchicine exposures: The Texas experience. South. Med. J..

[63] Premaratna R., Weerasinghe M.S., Premawardana N.P., de Silva H.J. (2015). Gloriosa superba poisoning mimicking an acute infection- a case report. BMC Pharmacol. Toxicol..

[64] Biçer S., Soysal D.D., Ctak A., Uçsel R., Karaböcüoğlu M., Uzel N. (2007). Acute colchicine intoxication in a child: A case report. Pediatr. Emerg. Care.

[65] Combalia A., Baliu-Piqué C., Fortea A., Ferrando J. (2016). Anagen effluvium following acute colchicine poisoning. Int. J. Trichol..

[66] Alaygut D., Kilic S.C., Kaya A., Oflaz M.B., Bolat F., Cevit Ö., Icagasioglu F.D. (2016). Assessment of 17 pediatric cases with colchicine poisoning in a 2-year period. Pediatr. Emerg. Care.

[67] Kim K., Shin M.K., Kim J.H., Kim M.H., Haw C.R., Park H.K. (2012). Effects of atopic dermatitis on the morphology and water content of scalp hair. Micros. Res. Tech..

[68] Plozzer C., Coletti C., Kokelj F., Trevisan G. (2000). Scanning electron microscopy study of hair shaft disorders in psoriasis. Acta Derm. Venereol. Suppl. (Stockh).

[69] Kumar B., Soni A., Saraswat A., Kaur I., Dogra S. (2008). Hair in psoriasis: A prospective, blinded scanning electron microscopic study. Clin. Exp. Dermatol..

[70] Shin M.K., Kim K.S., Ahn J.J., Kim N.I., Park H.K., Haw C.R. (2012). Investigation of the hair of patients with scalp psoriasis using atomic force microscopy. Clin. Exp. Dermatol..

[71] Kim K.S., Shin M.K., Ahn J.J., Haw C.R., Park H.K. (2013). A comparative study of hair shafts in scalp psoriasis and seborrheic dermatitis using atomic force microscopy. Skin Res. Technol..

[72] Pitney L., Weedon D., Pitney M. (2016). Is seborrhoeic dermatitis associated with a diffuse, low-grade folliculitis and progressive cicatricial alopecia?. Australas J. Dermatol..

[73] Dhaille F., Dillies A.S., Dessirier F., Reygagne P., Diouf M., Baltazard T., Lombart F., Hébert V., Chopinaud M., Verneuil L. (2019). A single typical trichoscopic feature is predictive of tinea capitis: A prospective multicentre study. Br. J. Dermatol..

[74] Mosley J.G., Gibbs A.C. (1996). Premature grey hair and hair loss among smokers: A new opportunity for health education?. BMJ.

[75] Chaudhry M., Ashraf T., Zeeshan N., Hanif A., Khan M., Ghazanfar I. (2018). Association of smoking with baldness and graying of hair among male adults. Biomedica.

[76] Babadjouni A., Pouldar Foulad D., Hedayati B., Evron E., Mesinkovska N. (2021). The effects of smoking on hair health: A systematic review. Skin Appendage Disord..

[77] Thom E. (2016). Stress and the hair growth cycle: Cortisol-induced hair growth disruption. J. Drugs Dermatol..

[78] Tosti A., Duque-Estrada B. (2009). Treatment strategies for alopecia. Expert Opin. Pharmacother..

[79] Diani A.R., Mulholland M.J., Shull K.L., Kubicek M.F., Johnson G.A., Schostarez H.J., Brunden M.N., Buhl A.E. (1992). Hair growth effects of oral administration of finasteride, a steroid 5-alpha reductase inhibitor, alone and in combination with topical minoxidil in the balding stump-tail macaque. J. Clin. Endocrinol. Metab..

[80] Olsen E.A., Hordinsky M., Whiting D., Stough D., Hobbs S., Ellis M.L., Wilson T., Rittmaster R.S., Dutasteride Alopecia Research Team (2006). The importance of dual 5 alpha redutase inhibition in the treatment of male pattern hair loss: Results of a randomized placebo-controlled study of dutasteride versus finasteride. J. Am. Acad. Dermatol..

[81] Vexiau P., Chaspoux C., Boudou P. (2002). Effects of minoxidil 2% vs. cyproterone acetate treatment on female androgenetic alopecia: A controlled, 12-month randomized trial. Br. J. Dermatol..

[82] Olsen E.A., Hordinsky M., Roberts J.L., Whiting D.A., Dermatologic Consortium for Women’s Health (2002). Female pattern hair loss. J. Am. Acad. Dermatol..

[83] Iorizzo M., Vincenzi C., Voudouris S., Piraccini B.M., Tosti A. (2006). Finasteride treatment of female pattern hair loss. Arch. Dermatol..

[84] Kohler C., Tschumi K., Bodmer C., Schneiter M., Birkhaeuser M. (2007). Effect of finasteride 5 mg (Proscar) on acne and alopecia in female patients with normal serum levels of free testosterone. Gynecol. Endocrinol..

[85] Pekmezci E., Dundar C., Turkoglu M. (2018). Proprietary herbal extract downregulates the gene expression of IL-1α in HaCaT Cells: Possible implications against nonscarring alopecia. Med. Arch..

[86] Kang W., Park S., Choi D., Son B., Park T. (2022). Activation of cAMP signaling in response to α-Phellandrene promotes vascular endothelial growth factor levels and proliferation in human dermal papilla cells. Int. J. Mol. Sci..

[87] Himaniarwati, Arba M., Susilawati Y., Mustarichie R. (2022). Hair growth promoting activity of Langir bark (*Albizia saponaria* Lour.) ethanol extract: In vivo assay. Rasayan J. Chem..

[88] León F., Hernandez-Zapata V., Bacab M.C., Maldonado G., Lezama J.A., Monteon V. (2020). The wound healing action of a cream latex formulation of *Jatropha gaumeri* Greenm. in a pre-clinical model. Vet. World.

[89] Marcovici G., Bauman A. (2020). An uncontrolled case series using a botanically derived, β-cyclodextrin inclusion complex in two androgenetic alopecia-affected male subjects. Cosmetics.

[90] Skowrońska W., Granica S., Dziedzic M., Kurkowiak J., Ziaja M., Bazylko A. (2021). *Arctium lappa* and *Arctium tomentosum*, sources of *Arctii radix*: Comparison of anti-lipoxygenase and antioxidant activity as well as the chemical composition of extracts from aerial parts and from roots. Plants.

[91] Kim B.H., Lee M.J., Lee W.Y., Pyo J., Shin M.S., Hwang G.S., Shin D., Kim C.E., Park E.S., Kang K.S. (2021). Hair growth stimulation effect of *Centipeda minima* extract: Identification of active compounds and anagen-activating signaling pathways. Biomolecules.

[92] Madhunithya E., Venkatesh G., Shyamala G., Manjari V., Ramesh S., Karuppaiah A., Sankar V. (2021). Development of ethosome comprising combined herbal extracts and its effect on hair growth. Adv. Trad. Med..

[93] Serruya R., Maor Y. (2021). Hair growth-promotion effects at the cellular level and antioxidant activity of the plant-based extract Phyllotex™. Heliyon.

[94] Ruksiriwanich W., Khantham C., Muangsanguan A., Chittasupho C., Rachtanapun P., Jantanasakulwong K., Phimolsiripol Y., Sommano S.R., Sringarm K., Ferrer E. (2022). Phytochemical constitution, anti-inflammation, anti-androgen, and hair growth-promoting potential of shallot (*Allium ascalonicum* L.) extract. Plants.

[95] Hyun J., Im J., Kim S.-W., Kim H.Y., Seo I., Bhang S.H. (2021). *Morus alba* root extract induces the anagen phase in the human hair follicle dermal papilla cells. Pharmaceutics.

[96] Im J., Hyun J., Kim S.W., Bhang S.H. (2022). Enhancing the angiogenic and proliferative capacity of dermal fibroblasts with mulberry (*Morus alba* L) root extract. Tissue Eng. Regen. Med..

[97] Galkin A.Y., Solovjova V.F., Dugan A.M. (2013). Anti-inflammatory and immunomodulating properties of the herbal preparation indicated for prevention and treatment of alopecia. Botanics.

[98] Park S.O., Park B.S., Noh G.Y. (2015). Action mechanism of natural plant extracts for hair loss prevention and hair growth promotion in C57BL/6 Mice. Int. J. Pharmacol..

[99] Susanti L., Mustarichie R., Halimah E., Kurnia D., Setiawan A., Maladan Y. (2022). Anti-alopecia activity of alkaloids group from noni fruit against dihydrotestosterone-induced male rabbits and its molecular mechanism: In vivo and in silico studies. Pharmaceuticals.

[100] Joshi P.S., Patil Y.B., Nagarkar B., Paul T.S., Apte K.G. (2021). In vivo phytotherapy in BALB/c athymic nude mice: Hair growth promotion using *Ficus religiosa* L. and *Morus alba* L.. J. Nat. Remedies.

[101] Park S.O., Park B.S., Noh G.Y. (2014). Effect of natural plant extract (Abelmo) on action mechanism and hair growth activities in C57BL/6 mice. J. Korean Oil chem. Soc..

[102] Rao G.V., Mukhopadhyay T., Ranganathan S., Madhavi M.S.L., Annamalai T., Lavakumar S. (2013). Chemical examination of three Indian medicinal plants and their hair growth evaluation studies. Arch. Appl. Sci. Res..

[103] Rusu M., Csedo C., Marcus G., Lupuliasa D. (2008). Preclinical study on the hair growth and regeneration of external use lotions containing castor oil (*Ricini oleum*) in rabbits. Farmacia.

[104] Kporou E., Sitapha O., Moussa G., Gouedji Y., Kra A., Djaman J. (2021). Quality, safety and activity of an ointment formulated from *Butyrospermum parkii* and *Ricinus communis* oils on rabbits hair growth. Rev. RAMReS-Ser. Pharm. Med. Trad. Afr..

[105] Tiwari R., Tiwari G., Yadav A., Ramachandran V. (2021). Development and evaluation of herbal hair serum: A traditional way to improve hair quality. Open Dermatol. J..

[106] Begum S., Lee M.R., Gu L.J., Hossain M.J., Kim H.K., Sung C.K. (2014). Comparative hair restorer efficacy of medicinal herb on nude (Foxn1nu) mice. BioMed Res. Int..

[107] Kumar N., Chaiyasut C. (2015). Hair growth promoting activity of *Carthamus tinctorius* florets extract-loaded nanostructured lipid carriers. Int. J. Pharm. Pharm. Sci..

[108] Imtiaz F., Islam M., Saeed H., Saleem B., Asghar M., Saleem Z. (2017). Impact of *Trigonella foenum*-*graecum* leaves extract on mice hair growth. Pakistan J. Zool..

[109] Dahmani M.M., Laoufi R., Selama O., Arab K. (2018). Gas chromatography coupled to mass spectrometry characterization, anti-inflammatory effect, wound-healing potential, and hair growth-promoting activity of Algerian *Carthamus caeruleus* L. (*Asteraceae*). Indian J. Pharmacol..

[110] Trivedi R.V., Bansod P.G., Taksande J.B., Mahore J.G., Tripurneni S.R., Rai K.R., Umekar M.J. (2019). Investigation of hair growth promoting ability of herbal gel containing *Zingiber officinale*. Int. J. Res. Pharm. Sci..

[111] Sakib S.A., Tareq A.M., Islam A., Rakib A., Islam M.N., Uddin M.A., Rahman M.M., Seidel V., Emran T.B. (2021). Anti-inflammatory, thrombolytic and hair-growth promoting activity of the *n*-hexane fraction of the methanol extract of *Leea indica* leaves. Plants.

[112] Choi H.C., Nam G.W., Jeong N.H., Choi B.Y. (2019). Hair growth promotion by extracts of *Inula Helenium* and *Caesalpinia sappan* bark in patients with androgenetic alopecia: A pre-clinical study using phototrichogram analysis. Cosmetics.

[113] Ablon G. (2015). A 3-month, randomized, double-blind, placebo-controlled study evaluating the ability of an extra-strength marine protein supplement to promote hair growth and decrease shedding in women with self-perceived thinning hair. Dermatol. Res. Pract..

[114] Ablon G., Kogan S. (2021). A randomized, double-blind, placebo-controlled study of a nutraceutical supplement for promoting hair growth in perimenopausal, menopausal, and postmenopausal women with thinning hair. J. Drugs Dermatol..

[115] Ablon G., Kogan S. (2018). A six-month, randomized, double-blind, placebo-controlled study evaluating the safety and efficacy of a nutraceutical supplement for promoting hair growth in women with self-perceived thinning hair. J. Drugs Dermatol..

[116] Kim B.H., Lee W.-Y., Trinh T.A., Pyo J.S., Lee S., Kim C.-E., Lee D.H., Park E.-S., Kang K.S. (2020). Hair growth effect of emulsion extracted brevilin A, a JAK3 inhibitor, from *Centipeda minima*. Processes.

[117] Sim J.B., Park S.O., Park B.S., Noh G.Y. (2016). Effect of natural plant extracts on hair loss prevent in people with alopecia. Asian J. Dermatol..

[118] Shin S., Kim K., Lee M.J., Lee J., Choi S., Kim K.S., Ko J.M., Han H., Kim S.Y., Youn H.J. (2016). Epigallocatechin gallate-mediated alteration of the microRNA expression profile in 5-dihydrotestosterone-treated human dermal papilla cells. Ann. Dermatol..

[119] Fischer T.W., Herczeg-Lisztes E., Funk W., Zillikens D., Bıro T., Paus R. (2014). Differential effects of caffeine on hair shaft elongation, matrix and outer root sheath keratinocyte proliferation, and transforming growth factor-beta2/insulin-like growth factor- 1-mediated regulation of the hair cycle in male and female human hair follicles in vitro. Br. J. Dermatol..

[120] Ehsani A.H., Toosi S., Seirafi H., Akhyani M., Hosseini M., Azadi R., Noormohamadpour P., Ghanadan A. (2009). Capsaicin vs. clobetasol for the treatment of localized alopecia areata. J. Eur. Acad. Dermatol. Venereol..

[121] Ablon G. (2016). A 6-month, randomized, double-blind, placebo-controlled study evaluating the ability of a marine complex supplement to promote hair growth in men with thinning hair. J. Cosmet. Dermatol..

[122] Sharquie K.E., Al-Obaidi H.K. (2002). Onion juice (*Allium cepa* L.), a new topical treatment for alopecia areata. J. Dermatol..

[123] Takahashi T., Kamimura A., Yokoo Y., Honda S., Watanabe Y. (2001). The first clinical trial of topical application of procyanidin B-2 to investigate its potential as a hair growing agent. Phytother. Res..

[124] Cho Y.H., Lee S.Y., Jeong D.W., Choi E.J., Kim Y.J., Lee J.G., Yi Y.H., Cha H.S. (2014). Effect of pumpkin seed oil on hair growth in men with androgenetic alopecia: A randomized, double-blind, placebo-controlled trial. Evid.-Based Complement. Altern. Med..

[125] Park G.H., Park K.Y., Cho H.I., Lee S.M., Han J.S., Won C.H., Chang S.E., Lee M.W., Choi J.H., Moon K.C. (2015). Red ginseng extract promotes the hair growth in cultured human hair follicles. J. Med. Food..

[126] Wessagowit V., Tangjaturonrusamee C., Kootiratrakarn T., Bunnag T., Pimonrat T., Muangdang N., Pichai P. (2016). Treatment of male androgenetic alopecia with topical products containing *Serenoa repens* extract. Australas J. Dermatol..

[127] Fischer T.W., Hipler U.C., Elsner P. (2007). Effect of caffeine and testosterone on the proliferation of human hair follicles in vitro. Int. J. Dermatol..

[128] Dhurat R., Chitallia J., May T.W., Jayaraaman A.M., Madhukara J., Anandan S., Vaidya P., Klenk A. (2017). An open-label randomized multicenter study assessing the noninferiority of a caffeine based topical liquid 0.2% versus minoxidil 5% solution in male androgenetic alopecia. Skin Pharmacol. Physiol..

[129] Hamed F.N., McDonagh A.J.G., Almaghrabi S., Bakri Y., Messenger A.G., Tazi-Ahnini R. (2018). Epigallocatechin-3 gallate inhibits STAT-1/JAK2/IRF-1/HLA-DR/HLAB and reduces CD8 MKG2D lymphocytes of alopecia areata patients. Int. J. Environ. Res. Public Health.

[130] Shin H., Cho A.R., Kim D.Y., Munkhbayer S., Choi S.J., Jang S., Kim S.H., Shin H.C., Kwon O. (2016). Enhancement of human hair growth using *Ecklonia cava* Polyphenols. Ann. Dermatol..

[131] Rastegar H., Ashtiani H.A., Aghaei M., Barikbin B., Ehsani A. (2015). Herbal extracts induce dermal papilla cell proliferation of human hair follicles. Ann. Dermatol..

[132] Pekmezci E., Dundar C., Turkoglu M. (2018). A proprietary herbal extract against hair loss in androgenetic alopecia and telogen effluvium: A placebo-controlled, single-blind, clinical-instrumental study. Acta Dermatovenerol. Alp. Pannonica Adriat..

[133] Rossi A., Mari E., Scarno M., Garelli V., Maxia C., Scali E., Iorio A., Carlesimo M. (2012). Comparative effectiveness of finasteride vs Serenoa repens in male androgenetic alopecia: A two-year study. Int. J. Immunopathol. Pharmacol..

[134] Prager N., Bickett K., French N., Marcovici G. (2002). A randomized, double-blind, placebo-controlled trial to determine the effectiveness of botanically derived inhibitors of 5-alpha-reductase in the treatment of androgenetic alopecia. J. Altern. Complement. Med..

[135] Li Z., Ryu S.W., Lee J., Choi K., Kim S., Choi C. (2016). Protopanaxatirol type ginsenoside Re promotes cyclic growth of hair follicles via inhibiting transforming growth factor β signaling cascades. Biochem. Biophys. Res. Commun..

[136] Hibino T., Nishiyama T. (2004). Role of TGF-beta2 in the human hair cycle. J. Dermatol. Sci..

[137] Suzuki A., Matsuura D., Kanatani H., Yano S., Tsunakawa M., Matsuyama S., Shigemori H. (2017). Inhibitory effects of polyacetylene compounds from *Panax ginseng* on neurotrophin receptor-mediated hair growth. Biol. Pharm. Bull..

[138] Shin D.H., Cha Y.J., Yang K.E., Jang I.S., Son C.G., Kim B.H., Kim J.M. (2014). Ginsenoside rg3 up-regulates the expression of vascular endothelial growth factor in human dermal papilla cells and mouse hair follicles. Phytother. Res..

[139] Lee Y., Kim S.N., Hong Y.D., Park B.C., Na Y. (2017). Panax ginseng extract antagonizes the effect of DKK1-induced catagen-like changes of hair follicles. Int. J. Mol. Med..

[140] Shin H.S., Park S.Y., Hwang E.S., Lee D.G., Song H.G., Mavlonov G.T., Yi T.H. (2014). The inductive effect of ginsenoside F2 on hair growth by altering the Wnt signal pathway in telogen mouse skin. Eur. J. Pharmacol..

[141] Truong V.L., Bak M.J., Lee C., Jun M., Jeong W.S. (2017). Hair regenerative mechanisms of red ginseng oil and its major components in the testosterone-induced delay of anagen entry in C57BL/6 mice. Molecules.

[142] Wei Y., Huyghues-Despointes B.M., Tsai J., Scholtz J.M. (2007). NMR study and molecular dynamics simulations of optimized beta-hairpin fragments of protein G. Proteins.

[143] Park S.H., Seo W., Eun H.S., Kim S.Y., Jo E., Kim M.H., Choi W.M., Lee J.H., Shim Y.R., Cui C.H. (2016). Protective effects of ginsenoside F2 on 12-O-tetradecanoylphorbol-13-acetate-induced skin inflammation in mice. Biochem. Biophys. Res. Commun..

[144] Andl T., Reddy S.T., Gaddapara T., Millar S.E. (2002). Wnt signals are required for the initiation of hair follicle development. Dev. Cell.

[145] Paus R., Ito N., Takigawa M., Ito T. (2003). The hair follicle and immune privilege. J. Investig. Dermatol. Symp. Proc..

[146] Xing L., Dai Z., Jabbari A., Cerise J.E., Higgins C.A., Gong W., de Jong A., Harel S., DeStefano G.M., Rothman L. (2014). Alopecia areata is driven by cytotoxic t lymphocytes and is reversed by Jak inhibition. Nat. Med..

[147] Ito T., Ito N., Saathoff M., Bettermann A., Takigawa M., Paus R. (2005). Interferon-gamma is a potent inducer of catagen-like changes in cultured human anagen hair follicles. Br. J. Dermatol..

[148] Bratty M.A., Alhazmi H.A., Thangavel N. (2021). GC–MS profiling and in silico prediction of MAPK receptor activation by fatty acids of watercress oil for hair growth marketed in Saudi Arabia. J. Saudi Chem. Soc..

